# Looking at the gaps and program needs to address the impact on children of agricultural workers in Puerto Rico during and after public health emergencies

**DOI:** 10.3389/fpubh.2022.1046701

**Published:** 2022-11-07

**Authors:** Marysel Pagán-Santana, Amy K. Liebman, Aníbal Y. López-Correa

**Affiliations:** ^1^Migrant Clinicians Network, Puerto Rico Office, San Juan, PR, United States; ^2^Migrant Clinicians Network, Environmental and Occupational Health, Salisbury, MD, United States

**Keywords:** Puerto Rico, farmworkers, children, climate, emergencies

## Introduction

Hurricanes Irma and Maria in the Caribbean, wildfires in the western United States, flooding in parts of Pakistan, and extreme heat waves in Europe are examples of recent natural disasters that became public health emergencies. The consequences of these events, including access to essential services and damage and destruction of infrastructure, create a cascading impact, affecting the immediate and long-term health and well-being of the population. Intense climate-related events are projected to increase in intensity and impact (1). However, these do not affect everyone equally and vary depending on the characteristics of individuals, their work, ethnicity, residence, and language, among others (2, 3). Moreover, the social, economic, and political systems in which these events occur can function as either increase risk or foster protection.

In the case of agricultural workers, they are subject to various environmental stressors throughout their workday and experience greater risks than workers in most other industries (4). They are in a more vulnerable position regarding the effects that climate change may have on their work. Moreover, other psychosocial factors intrinsic to farming, such as social and working conditions, made agricultural workers one of the most affected populations during the COVID-19 pandemic (5). The physical effects that extreme events can have on the health of agricultural workers, and the damage and social effects of public health emergencies related to the climate crisis can also impact their children, especially those who are also part of the agricultural labor force. For minors who participate in agricultural work, the effect of disasters adds to the damage and general impact of high-risk exposures and lack of labor protection that are already of concern because of the effect on their health and development.

In addition to the characteristics and vulnerabilities of individuals, there are geographic regions where the risk and vulnerability to extreme climate-related is higher. This is the case of Puerto Rico, which has experienced several significant climate-related events in the past 10 years that caused millions of dollars in economic losses and the deaths of thousands of people. These catastrophic events have placed Puerto Rico first among the places most affected by the climate, according to the 2021 Global Climate Risk Index report (6). Beyond these climatic threats, this US territory faces economic and political challenges weakening potential protective factors during and after these disasters. This includes limited access to education and health services and essential infrastructures such as water, electricity, and communications. Housing and food security are also impacted. When considering these geographic, environmental, and political factors, as well as the socio-economic indicators of individuals, some populations are extremely vulnerable, which is the case of farming communities in Puerto Rico.

## Castañer and the historic impact of disasters

Castañer, Puerto Rico, exemplifies an agricultural region that natural and public health disasters have greatly impacted. This mountainous farming community is in the west central region of Puerto Rico between the municipalities of Yauco, Lares, Adjuntas, and Maricao. The region is a key agricultural area due to a large amount of land and crops. The labor structure of the agricultural industry in Puerto Rico is extremely dynamic. Many families have small family farms or “fincas” and often the entire family, including the children, participate in its productivity in some fashion. At the same time, many of these workers are employed on larger, corporately owned farms. In 2012, the Castañer region had about 2,574 farms and 1,428 agricultural workers over 16 years of age (7, 8). The predominant crops include coffee, orange, banana, and plantain, mostly from small or family farms. More than 50% of people in this region are below the federal poverty level, 95% are Hispanic/Latino, and the average age is between 42 and 44 years (8). The area is no stranger to catastrophes, such as Hurricane Maria in 2017 and Hurricane Fiona in 2022, that have significatively affected the island. These natural events add additional stress factors to a major economic and migratory crisis Puerto Rico has been experiencing for over a decade. Castañer is highly susceptible to landslides and is among the regions that reported more than 25 landslides per square kilometer during Hurricane Maria. About 74 residential areas were marked as flooding risk (9, 10). Fiona was a category one hurricane that left over 30 inches of rain in some regions in 72 h period, significantly impacting agriculture, the stability of the education system, electricity, and services (11). Additional public health concerns have been documented due to this hurricane, including lack of safe water, increased risk factors for respiratory or cardiovascular diseases, and exposure to infectious diseases such as leptospirosis (12, 13).

In understanding the impact of public health emergencies on the children of agricultural workers, Castañer offers an important example of compounding and cascading events that affected this population. Climate-related disasters and the COVID-19 pandemic impacted the health and well-being of children. The livelihoods of agricultural families were affected due to substantial crop loss, destroyed or damaged homes, lack of resources, food insecurity, and interrupted education. When looking at the profile of children in Puerto Rico, about 57% of children live below the federal poverty level and an estimated 23% are likely to suffer from food insecurity (14, 15). The amount is substantially greater in the Castañer region, reaching between 60 and 82% of children living in poverty (14). There is one federally qualified community health center that offers services to nearly 11,279 patients in the region. Of those patients, 24% are under 18 years of age, 95% are below the poverty level, and 25% are identified as farmers and dependents of farmers (16). Prevalent childhood health conditions in the region include asthma and obesity (17, 18). In terms of educational services, this region has faced the closure or relocation of schools, which reduces immediate access to education and other associated services such as meals. In a region with a school desertion rate that can be as high as 6% compared to 2.9% for the territory, this presents a major threat to the social and economic mobility of the region (19). Furthermore, traveling distance to the nearest schools can take more than 30 min in these mountainous areas, which represents a significant burden for a population with such high levels of poverty. These social and environmental spheres must be considered when we analyze the impact of public health emergencies in the region, such as COVID-19.

During the beginning of the COVID-19 pandemic in 2020, schools in Puerto Rico and other related services were shut down for several months. In August 2020, the Puerto Rico Department of Education implemented remote learning, requiring access to internet services, consistent electricity service, and access to computers. Several reports document the exacerbated situation that students in rural areas experienced regarding education, including low turnout or students not reporting to classes due to limitations in technology, as well as challenges of parents finding childcare during work hours. As a sphere of social protection, the absence of school services, in turn, implied limitations in food security and physical security. During this period, Puerto Rico registered increased maltreatment due to negligence (20). The in-person return to class in Puerto Rico was driven by the observation that the state educational system had struggled to reach some rural populations and that these areas were at a substantial disadvantage in terms of academic achievement. However, the in-person return in the Castañer region was affected by several factors, including schools under repair due to Hurricane Maria or the southern earthquakes, schools closed for economic reasons, and/or levels of transmission of COVID-19 reported for the region. Schools in this region reported a significant number of outbreaks and a high percentage of positivity of COVID-19 cases among students (21). The region had limited vaccination and testing services, with three vaccination centers and one center for testing or free medical care for the pediatric population.

## Discussion

Even though there are reports documenting the impact of public health emergencies in Puerto Rico, very few explore vulnerable populations such as agricultural communities. This contrasts with recovery efforts aiming at maximizing agricultural production and social development and mobilization in rural areas. For Castañer, while a large part of the population participates in the agricultural industry, the response and recovery initiatives are falling short of improving social conditions. Although there are no specific studies examining the impact of these emergencies on agricultural worker children in Puerto Rico, given the described poverty levels and education limitations, it is highly likely that they have been greatly impacted by recent public health emergencies. We explored various publications and statistical reports to understand what the social and environmental conditions of children of agricultural communities are following 5 years of continuous emergencies and disasters. Table 1 presents the overall findings for the Castañer region. The lack of data on the impact of climate and public health emergencies on minors who are part of the agricultural community limits the implementation of programs that support their development and promote their social mobility while protecting their health.

**Table 1:** Characteristics and living conditions of children in agricultural communities in Puerto Rico before, during, and after emergencies.

**Emergencies/Disasters history**	Puerto Rico experienced five major disasters and emergencies in the last 5 years, including 2017 Hurricanes Irma and María, 2019–20 Southeast earthquakes. 2020–22 COVID-19, 2022 Drought, and 2022 Hurricane Fiona.
	**Impact on agricultural regions flooding and landslides was from significant to catastrophic**.
**Conditions of one agricultural community in Puerto Rico**	The island has a fragile utilities infrastructure (water, power, communications, roads).
	60–82% children in one agricultural region live in poverty
	Limited access to schools due to planned closures
	One community health center and three vaccination sites.
**Outcomes**	Children in the region experienced limited access to healthcare and 5 years of interrupted/limited education.
	Food, economic, and physical security was impacted
	Exposure to infectious diseases including COVID-19 and leptopirosis

## Conclusion

Understanding the needs of children in agricultural families, as well as exploring the impact of the social spheres that may affect their well-being, is crucial to developing and adapting programs that target their health and safety. Assessing the impact of emergencies on children's health is especially essential for regions like Puerto Rico, with a higher risk of natural disasters and more intense disasters due to the climate crisis. Puerto Rico, its agricultural communities and the children in these regions should be prioritized.

## Author contributions

All authors contributed to the discussion, research, and analysis of the reports and to the writing of the manuscript. All authors contributed to the article and approved the submitted version.

## Funding

This work was supported by the National Institute for Occupational Safety and Health under Grant U54 OH009568-10 and Marshfield Clinic Research Institute.

## Conflict of interest

The authors declare that the research was conducted in the absence of any commercial or financial relationships that could be construed as a potential conflict of interest.

## Publisher's note

All claims expressed in this article are solely those of the authors and do not necessarily represent those of their affiliated organizations, or those of the publisher, the editors and the reviewers. Any product that may be evaluated in this article, or claim that may be made by its manufacturer, is not guaranteed or endorsed by the publisher.
